# Mitochondrial Cardiomyopathy Presenting as Dilated Phase of Hypertrophic Cardiomyopathy Diagnosed with Histological and Genetic Analyses

**DOI:** 10.1155/2017/9473917

**Published:** 2017-05-23

**Authors:** Toshiki Kuno, Syohei Imaeda, Yohei Asakawa, Hiroshi Nakamura, Genzou Takemura, Daisuke Asahara, Akira Kanamori, Tomoyuki Kabutoya, Yohei Numasawa

**Affiliations:** ^1^Department of Cardiology, Japanese Red Cross Ashikaga Hospital, Ashikaga, Japan; ^2^Department of Neurology, Japanese Red Cross Ashikaga Hospital, Ashikaga, Japan; ^3^Department of General Internal Medicine, Hiroshima-Nishi Medical Center, Ohtake, Japan; ^4^Department of Internal Medicine, Asahi School of Dentistry University, Mizuho, Japan; ^5^Department of Gastroenterology, Japanese Red Cross Ashikaga Hospital, Ashikaga, Japan; ^6^Department of Cardiology, Jichi Medical University, Shimotsuke, Japan

## Abstract

We report a case with 46-year-old man diagnosed with mitochondrial cardiomyopathy in the dilated phase of hypertrophic cardiomyopathy. Since cardiac magnetic resonance imaging, beta-methyl-p-^123^I-iodophenyl-pentadecanoic myocardial scintigraphy, and positron emission tomography/computed tomography revealed no remarkable findings, we performed electron microscopic examination, which aided in diagnosing mitochondrial cardiomyopathy. Muscle biopsy was also compatible with mitochondrial encephalomyopathy, lactic acidosis, and stroke-like episodes and DNA analysis also concluded it. Since muscle biopsy is less invasive for patients compared to endomyocardial biopsy, cardiologists need to consider it. The diagnosis of mitochondrial cardiomyopathy is helpful because it is a genetic condition and also for consideration of device therapy, as well as management for acute crisis.

## 1. Introduction

Mitochondrial disease is a heterogeneous group of multisystemic diseases due to mutations in nuclear or mitochondrial DNA. Although multimodalities aid the diagnosis, the diagnosis of mitochondrial cardiomyopathy is challenging because histological analysis is sometimes needed [1–3]. We report a case with 46-year-old man diagnosed with mitochondrial cardiomyopathy in the dilated phase of hypertrophic cardiomyopathy diagnosed with muscle and endomyocardial biopsy and genetic analyses.

## 2. Case Report

**Figure 1:**
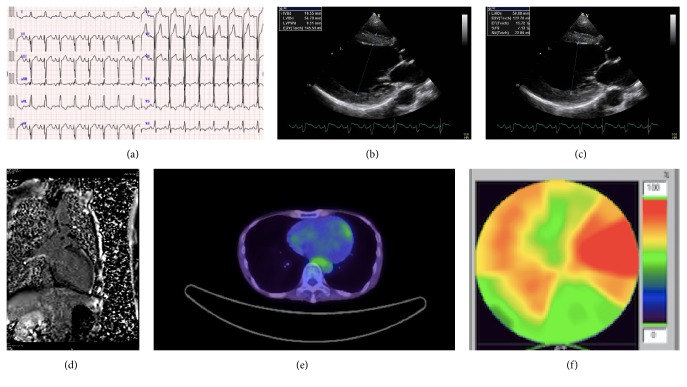
(a) Electrocardiogram showed left ventricular hypertrophy with ST changes. (b) Transthoracic echocardiography, long axis view, end-diastolic phase, left ventricular end-diastolic diameter = 54 mm, interventricular septal diameter = 10.6 mm, and left ventricular posterior wall diameter = 9.5 mm. (c) Transthoracic echocardiography, long axis view, end-systolic phase, and left ventricular end-systolic diameter = 48 mm. (d) Magnetic resonance imaging showed no delayed enhancement. (e) Positron emission tomography-computed tomography showed no remarkable findings. (f) Beta-methyl-p-^123^I-iodophenyl-pentadecanoic (^123^I-BMIPP) myocardial scintigraphy showed diffuse decreased accumulation except lateral wall.

**Figure 2:**
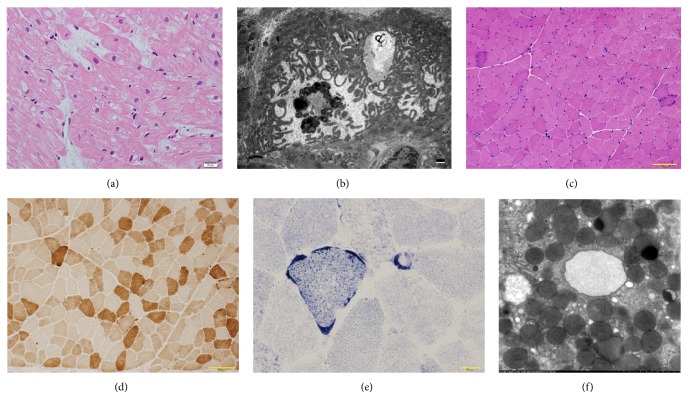
(a) Light micrographs from endomyocardial biopsy revealed vacuolar change in myocardial cells, especially around nucleus and perimysial fibrosis without inflammatory cells (hematoxylin eosin stain, bar: 20 *μ*m). (b) Electron microscopic examination of the endomyocardial biopsy revealed mitochondrial deformations with various types (e.g., sausage-like, loop-like, small, or gigantic), bar: 1 *μ*m. (c) Light micrographs of the muscle biopsy revealed ragged red fibers (hematoxylin eosin stain, bar: 100 *μ*m). (d) Light micrographs of the muscle biopsy revealed decreased cytochrome c oxidase (COX) activity (COX stain, bar: 100 *μ*m). (e) Light micrographs of the muscle biopsy revealed blood vessels strongly reactive (SSV) to succinate dehydrogenase (SDH) (SDH stain, bar: 100 *μ*m). (f) Electron microscopic examination of gastric biopsy revealed normal mitochondria (×1,2000).

A 46-year-old man visited an outpatient clinic complaining of appetite loss since a month ago. He was lean with short stature, weighing 31 kg, and 157 cm tall. He had been diagnosed with hypertrophic cardiomyopathy at the age of 40 years and had sensory hearing loss and diabetes mellitus. An echocardiography one year ago showed diffuse left ventricular hypertrophy (interventricular-septal wall thickness: 12 mm, lateral wall thickness: 14 mm) with an ejection fraction of 52%. His mother had died at the age of 50 due to dilated cardiomyopathy and had a history of diabetes mellitus. His brother also suffered hearing loss. The patient was a nonsmoker with no history of hypertension. His electrocardiogram revealed normal sinus rhythm and complete left bundle branch block (QRS duration: 128 ms) (Figure 1(a)). B-type natriuretic peptide was 176 pg/mL and troponin-T level was 0.18 ng/mL. Echocardiography revealed severely compromised global left ventricular systolic function, except the lateral wall, with an ejection fraction of 20% (Figures 1(b) and 1(c), Online video supplement 1, Supplementary Material available online at https://doi.org/10.1155/2017/9473917). Serum lactate and pyruvate levels and cerebrospinal fluid lactate and pyruvate levels were elevated (24.4, 1.2, and 41.8, 1.4 mg/dL, resp.). Cardiac magnetic resonance imaging (MRI) showed no late gadolinium enhancement (LGE); positron emission tomography/computed tomography (PET-CT) showed no remarkable findings (Figures 1(d) and 1(e)). Beta-methyl-p-^123^I-iodophenyl-pentadecanoic (^123^I-BMIPP) myocardial scintigraphy revealed diffuse reduced uptake, except on the lateral wall (Figure 1(f)). Coronary angiography showed intact arteries. Endomyocardial biopsy revealed vacuolar changes in the myocardial cells (especially around nucleus) and perimysial fibrosis without inflammatory cells (Figure 2(a)). Electron microscopic examination showed increased numbers of mitochondria with various types of deformations, differences in size, and rare fraction of myofibril (Figure 2(b)). Since the patient was quite lean, we performed a muscle biopsy, which revealed ragged red fibers without cytochrome c oxidase activity, which is compatible with mitochondrial myopathy (Figures 2(c)–2(e)). Finally, mitochondrial DNA analysis of his blood sample showed m.3243A>G mutation and mitochondrial encephalomyopathy, lactic acidosis, and stroke-like episodes (MELAS) syndrome. However, he denied any symptoms such as weakness and his MRI of the head did not show any remarkable findings.

The diagnosis was concluded as mitochondrial cardiomyopathy and MELAS. After treatment with intravenous dobutamine for reduced output, he was given angiotensin-converting enzyme inhibitor, beta-blocker, spironolactone, coenzyme Q10, and carnitine. Since he complained of nausea after dobutamine therapy, we performed a gastroscopy and stomach biopsy. Electron microscopic examination showed no mitochondrial deformation (Figure 2(f)). At 7 months' follow-up after medical therapy, echocardiography revealed no change in systolic dysfunction and he had mild dyspnea on exertion (New York Heart Association, Class II). Troponin-T level remained elevated (0.18 ng/mL). He was then referred to another institute for the possibility of cardiac resynchronization and intracardiac defibrillation device therapy.

## 3. Discussion

We report a case, diagnosed with mitochondrial cardiomyopathy in the dilated phase of hypertrophic cardiomyopathy. Although 30% of cases of mitochondrial myopathy showed LGE, the MRI in our case revealed no significant findings in contrast to a previous study [2]. PET-CT in our case showed unremarkable findings as well. There is no published report of mitochondrial cardiomyopathy and PET-CT. Moreover, ^123^I-BMIPP myocardial scintigraphy revealed diffuse reduced uptake except in the lateral wall and fatty acid metabolism disturbances, but it was indefinite because another study insisted ^123^I-BMIPP hyperaccumulation is related to mitochondrial cardiomyopathy [3]. If we performed myocardial perfusion imaging, we would observe a metabolic-perfusion mismatch because of the intact coronary arteries. Since multimodal investigations revealed no conclusive findings, we performed electron microscopic examination, which aided in diagnosing mitochondrial cardiomyopathy [4]. Muscle biopsy was also compatible with MELAS and DNA analysis concluded MELAS. Since muscle biopsy is less invasive for patients compared to endomyocardial biopsy [5], cardiologists need to consider it. The diagnosis of mitochondrial cardiomyopathy as seen in our case is helpful because it is a genetic condition and also for consideration of device therapy, as well as management for acute crisis [6]. In our case, we could expect the response to CRT-D to decrease the risk of cardiac sudden death and heart failure symptoms, although cardiac MRI scan showed no LGE, which might suggest favorable outcomes [6–8].

## 4. Conclusion

We report a case of mitochondrial cardiomyopathy presenting as dilated phase of hypertrophic cardiomyopathy diagnosed with muscle and endomyocardial biopsy and genetic analyses. Since muscle biopsy is less invasive for patients compared to endomyocardial biopsy, cardiologists need to consider it regardless of muscle weakness.

## Supplementary Material

Online Video 1: Transthoracic echocardiography, long axis view.
Online Video 2: Transthoracic echocardiography, short axis view.
Online Video 3: Transthoracic echocardiography, 4 chambers view.
